# Evaluation of potential tissue heating during percutaneous drill-assisted bone sampling in an in vivo porcine study

**DOI:** 10.1007/s00256-021-03890-w

**Published:** 2021-08-30

**Authors:** Stefan M. Niehues, Sefer Elezkurtaj, Keno K. Bresssem, Bernd Hamm, Christoph Erxleben, Janis Vahldiek, Lisa C. Adams

**Affiliations:** 1 Department of Radiology, Charité University Berlin, Hindenburgdamm, 30, 12203 Berlin, Germany; 2 Department of Pathology, Charité University Berlin, Hindenburgdamm, 30, 12203 Berlin, Germany; 3 Department of Radiology, Charité - Universitätsmedizin Berlin, Corporate Member of Freie Universität Berlin, Humboldt-Universität Zu Berlin, and Berlin Institute of Health (BIH), Charitéplatz, 1, 10117 Berlin, Germany

**Keywords:** Battery-powered bone drilling, Bone temperature, Porcine model, Tissue damage

## Abstract

**Background:**

Minimally invasive, battery-powered drilling systems have become the preferred tool for obtaining representative samples from bone lesions. However, the heat generated during battery-powered bone drilling for bone biopsies has not yet been sufficiently investigated. Thermal necrosis can occur if the bone temperature exceeds a critical threshold for a certain period of time.

**Purpose:**

To investigate heat production as a function of femur temperature during and after battery-powered percutaneous bone drilling in a porcine in vivo model.

**Methods:**

We performed 16 femur drillings in 13 domestic pigs with an average age of 22 weeks and an average body temperature of 39.7 °C, using a battery-powered drilling system and an intraosseous temperature monitoring device. The standardized duration of the drilling procedure was 20 s. The bone core specimens obtained were embedded in 4% formalin, stained with haematoxylin and eosin (H&E) and sent for pathological analysis of tissue quality and signs of thermal damage.

**Results:**

No significant changes in the pigs’ local temperature were observed after bone drilling with a battery-powered drill device. Across all measurements, the median change in temperature between the initial measurement and the temperature measured after drilling (at 20 s) was 0.1 °C. Histological examination of the bone core specimens revealed no signs of mechanical or thermal damage.

**Conclusion:**

Overall, this preliminary study shows that battery-powered, drill-assisted harvesting of bone core specimens does not appear to cause mechanical or thermal damage.

## Introduction

Bone biopsies are obtained to determine the aetiology of bone lesions by subsequent histological examination. Minimally invasive, percutaneous image-guided biopsy techniques have become the preferred method for obtaining representative samples from bone lesions [1]. In recent years, a device for battery-powered drill-assisted bone tissue harvesting was introduced as a valuable alternative to conventional manual drilling [2–4]. Advantages of this battery-powered drill device include higher rotation speed with shorter intervention time, easier application and the possibility to collect larger core samples with less patient discomfort [3].

However, the heat generation during battery-powered single-hole bone drilling for bone sampling has not yet been sufficiently examined. Thermal necrosis has been reported in operative fracture treatment and reconstructive surgery due to an excessive increase in local temperature during extended drilling procedures [4]. If the temperature exceeds a critical threshold for a certain period of time, a denaturing process begins to develop in the tissue, which has a negative effect on the mechanical properties of the bone and may delay or even prevent subsequent bone healing [5]. No final consensus has yet been reached regarding the critical temperature threshold and duration of the procedure for thermal necrosis to occur [6].

With regard to battery-powered drill-assisted bone biopsies, it is currently unclear whether faster drilling with higher rotation speed will lead to greater heating of the needle and the adjacent bone and whether the resulting bone biopsies will show signs of thermal damage. Even during the heating of the battery-powered drilling needle, the tissue sample may be damaged because there is no heat sink effect within the needle.

Therefore, our aim was to investigate heat production as a function of bone temperature during and after battery-powered bone drilling. We used an in vivo porcine model to examine whether battery-powered drill-assisted bone tissue harvesting from the femur causes temperatures in the target tissue that could cause bone tissue damage.

## Materials and methods

### Animals, housing and care

At our facility, bone drillings (*n* = 16) were performed on 13 domestic pigs with an average age of 22 weeks. The animals were kept under controlled conditions in the stables of our facility and provided with food and drinking water. All procedures were performed in accordance with the local guidelines and rules for the implementation of the Animal Welfare Act. Before the drilling procedure, the pigs were narcotized and anaesthetized by an intramuscular and subsequently intravenous administration of ketamine, azaperone, xylazine and atropine as previously described by Vahldiek et al. [7]. The pigs received fentanyl as pain medication and a suprapubic catheter was placed in the bladder. Vital parameters (i.e. heart rate, oxygen saturation) were continuously monitored throughout the experiments by a veterinarian. The study and all interventions performed were carried out in accordance with the guidelines and rules of the Federation of Laboratory Animal Science Associations (FELASA) and approved by the National Office for Health and Social Affairs (LaGeSo, Berlin, Germany, G0281/12) and designed according to the ARRIVE guidelines.

### Temperature measurement during bone drilling

**Fig. 1:**
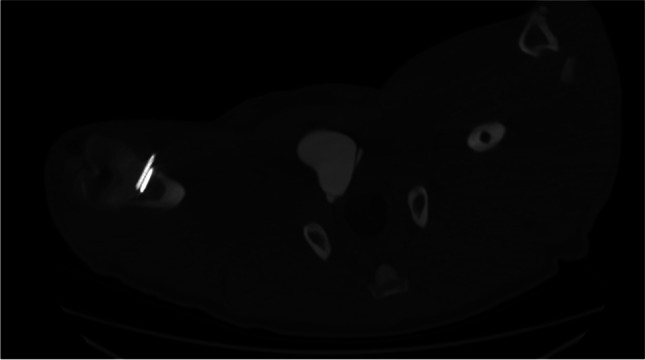
Illustration of the temperature monitoring device, advanced 3 cm into the bone, and the second drill placed directly beneath the measuring device

To ensure temperature monitoring, a hole was drilled percutaneously into the pig’s femur, using a powered bone biopsy device (Arrow® OnControl® Bone Lesion Biopsy System, Teleflex, Shavano Park, TX, USA). The FDA-cleared and CE-certified ARROW® OnControl® battery-powered bone access device is a sealed, hand-held, lithium battery-powered medical device with a driver and biopsy needle set. The needle set consists of two parts—an outer cannula with a length of 102 mm and a bevel-tipped inner stylet, which is used to penetrate the cortex. After removing the bone access needle, we advanced a calibrated 11-gauge temperature monitoring device (EN60751-normed PT-100 with an active 20-mm measuring zone at the needle tip) 3 cm into the bone (Fig. 1). A second drill was placed directly beneath the measuring device (Fig. 1). The median distance between the two probes was 0.96 mm (1.14 mm – 0.78 mm). During drilling, we continuously monitored and recorded the temperature of the tissue at 1-s intervals for a total of 60 s.

### Bone drilling procedure

**Fig. 2:**
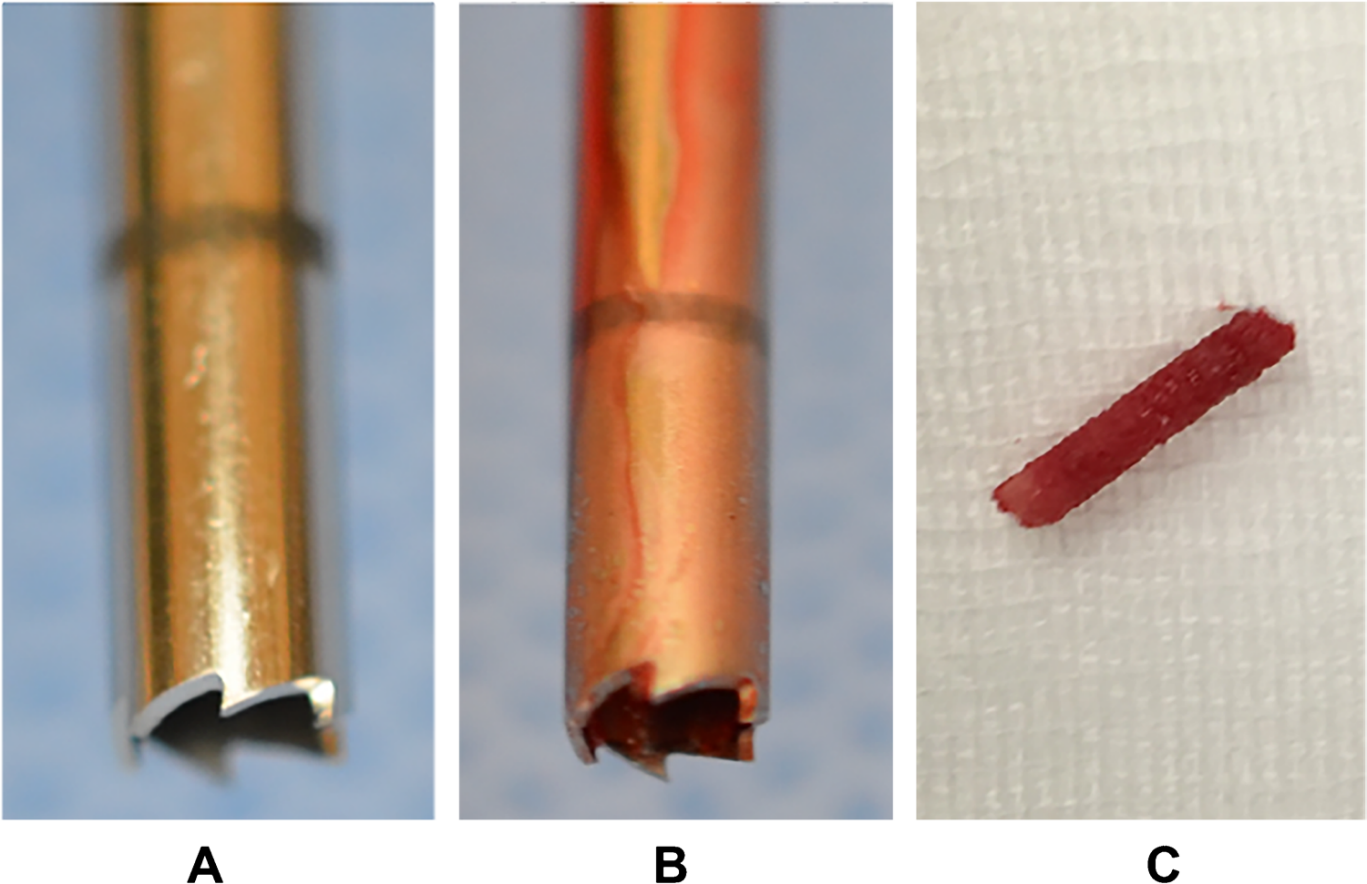
Illustration of the bone access needle before (A) and after drilling (B). (C) shows a sample from the femur bone

The bone drilling was performed using a battery-powered percutaneous drilling system (Arrow® OnControl® Bone Lesion Biopsy System, Teleflex, Shavano Park, TX, USA). This device operates at a single speed of approximately 420 rotations per minute. The 11-gauge bone access needle (Fig. 2) was inserted into the bone superficially next to and parallel to the previously inserted temperature monitoring probe. For bone tissue sampling, we used a 13-gauge bone lesion biopsy needle to harvest a 3-cm bone cylinder, following 20 s of active drilling in the pig’s femur. This is the drilling time recommended by the manufacturer for single procedures in the instructions for use. The correct parallel needle position and the correct drilling vector were visualized by CT fluoroscopy on an 80-slice multi-detector CT scanner (Aquilion PRIME, Canon Medical Systems, Otawara, Japan) (Fig. 1).

### Sample quality check and histological analysis

The bone core specimens obtained were embedded in 4% formalin and sent for pathological analysis of tissue quality and signs of thermal damage. For histological evaluation, the probes were decalcified with 10% ethylenediaminetetraacetic acid (EDTA) [8], conventionally dehydrated and embedded in paraffin wax. Finally, histological sections of 3–5 µm were prepared from the bone samples and stained with haematoxylin and eosin (H&E).

### Statistical analysis

The temperatures of each probe were recorded in the PT-100 device and digitally transferred as comma-separated values to a standard PC system. All statistical analysis was performed using “R” version 3.6.3 and the “tidyverse” library [9, 10]. Temperatures were not normally distributed and therefore expressed as medians and interquartile ranges (IQR). Ranges of temperature change were displayed as boxplots, while relative temperature changes over time compared to the initial measurement were displayed as line graphs applying a smoothing factor of 0.25. Temperature measurements were compared using the Wilcoxon rank-sum test. A *p*-value of < 0.05 was considered statistically significant.

## Results

A total of 15 of 16 in vivo femur drillings from 12 domestic pigs were included in the present analysis. One drilling was excluded from further analysis, because the temperature monitoring device was damaged by the drilling device during the drilling procedure, which lead to a distortion of the measured values. The quality and size of all core specimens (*n* = 15) classified as sufficient by a pathologist. There were no complications during the drilling procedures, all of which were performed with a standardized drilling time of exactly 20 s.

### Assessment of drill-related temperature changes within the porcine femur bone

**Fig. 3:**
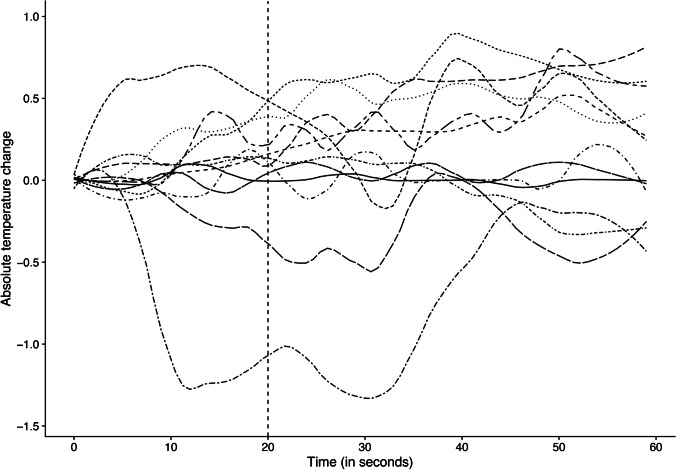
Temperature changes over a time interval of 60 s with each line representing one measurement (*n* = 15 in 12 pigs). Most temperature changes lie within ± 0.5 °C with no clear trend for heating or cooling after the end of the drilling procedure (at 20 s) or at the end of the measurement interval (at 60 s)

An overview of the measured temperatures in the porcine femora is given in Table 1. The median temperature of the pigs was 39.7 °C at the start of the measurements and 39.9 °C at the end of the measurements (at 60 s), corresponding to a normal porcine body temperature, which is approximately 39 °C with a range of up to 40 °C [11]. During the drilling process, we observed varying temperature changes across our 15 measurements without any significant trend (see Fig. 3). The observed variations in the collected individual measurements are likely due to differences in the body temperature of the pigs, variability in bone properties or deviations of the drilling path at initial contact.

**Table 1:** Overview of measured temperatures in the pigs’ femora (median, interquartile range, minimum, maximum), including the start temperature, the end temperature, the temperature difference between start temperature and temperature after drilling and the overall difference in temperature between start temperature and end temperature. *Abbreviations*: *n*, number; *IQR*, interquartile range; *Min.*, minimum; *Max.*, maximum; *°C*, temperature

*n*	Median	IQR	Min	Max	Max. change	Start °C	C° after drilling	End °C	°C difference after drilling	Overall °C difference
1	39.4	0.0	39.3	39.5	0.2	39.4	39.4	39.4	0.0	0.0
2	40.1	0.4	39.4	40.6	1.2	39.5	39.8	40.1	0.3	0.6
3	41.1	0.3	40.4	41.3	0.9	40.6	41.1	40.9	0.5	0.3
4	36.9	0.2	36.6	37.5	0.9	36.6	36.7	36.9	0.1	0.3
5	37.4	0.2	37.0	37.9	0.9	37.0	37.4	37.4	0.4	0.4
6	40.5	0.1	40.3	41.0	0.7	40.5	40.7	40.5	0.2	0.0
7	41.0	0.5	40.6	41.4	0.8	40.6	40.7	41.4	0.1	0.8
8	40.7	1.0	40.1	41.4	1.3	41.4	40.2	41.0	− 1.2	− 0.4
9	41.1	0.2	40.6	41.3	0.7	41.0	41.2	40.7	0.2	− 0.3
10	39.6	0.4	39.2	39.9	0.7	39.9	39.6	39.6	− 0.3	− 0.3
11	40.3	0.4	40.1	40.9	0.8	40.1	40.3	40.7	0.2	0.6
12	39.4	0.0	39.3	39.5	0.2	39.4	39.4	39.4	0.0	0.0
13	40.8	0.1	40.7	40.9	0.2	40.8	40.8	40.8	0.0	0.0
14	39.6	0.1	39.4	39.7	0.3	39.5	39.6	39.6	0.1	0.1
15	41.0	0.1	40.8	41.1	0.3	40.9	41.0	41.0	0.1	0.1

**Fig. 4:**
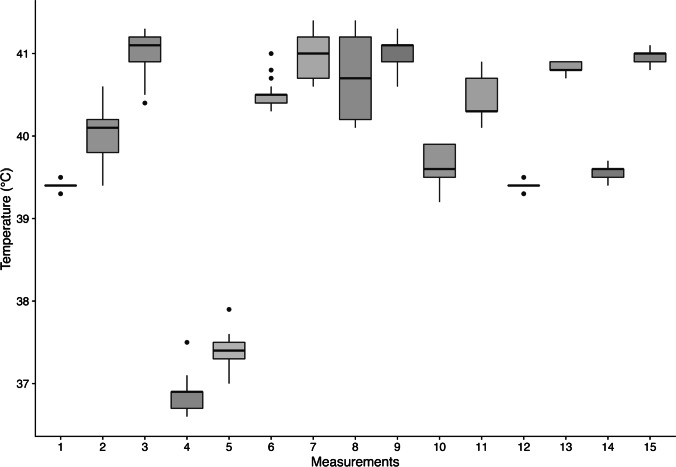
This figure shows boxplots for the range of temperature changes over the individual measurements (*n* = 15 in twelve domestic pigs). Each boxplot includes the minimum, the maximum, the sample median, and the first and third quartiles. Outliers are indicated by black dots

Figure 4 shows the corresponding boxplots for the range of temperature changes for all of our measurements. Each boxplot includes the minimum, the maximum, the sample median, and the first and third quartiles. A maximum change in temperature was observed in measurement number 8 with a maximum change of 1.2 °C. The median change in temperature (across all measurements) between the initial measurement and the temperature measured after drilling (at 20 s) was 0.1 °C and the median change in temperature between the initial and the final measurement after 60 s was also 0.1 °C. One of the pigs (measurements 4 and 5) had a lower body temperature of approximately 37 °C, which can be interpreted in the context of perioperative hypothermia due to an anaesthesia-related heat loss [12].

Across all measurements, no significant changes in temperature were found with a constant median temperature of 39.7 °C at the beginning of and after the drilling procedure (*p* = 0.84). Also, there was no significant difference between the end of the drilling procedure and the end of the temperature measurement after 60 s (*p* = 0.78) or between the first and last temperature measurement (*p* = 0.57). During the drilling process, the measured temperatures showed fluctuations of ± 0.5 °C compared to the initial measurement and started to fluctuate slightly more when the drilling was stopped after 20 s, but this trend was again not significant. Overall, we did not find any significant changes in the pigs’ temperature after bone drilling with a battery-powered drill device.

### Histological examination of the core specimens

**Fig. 5:**
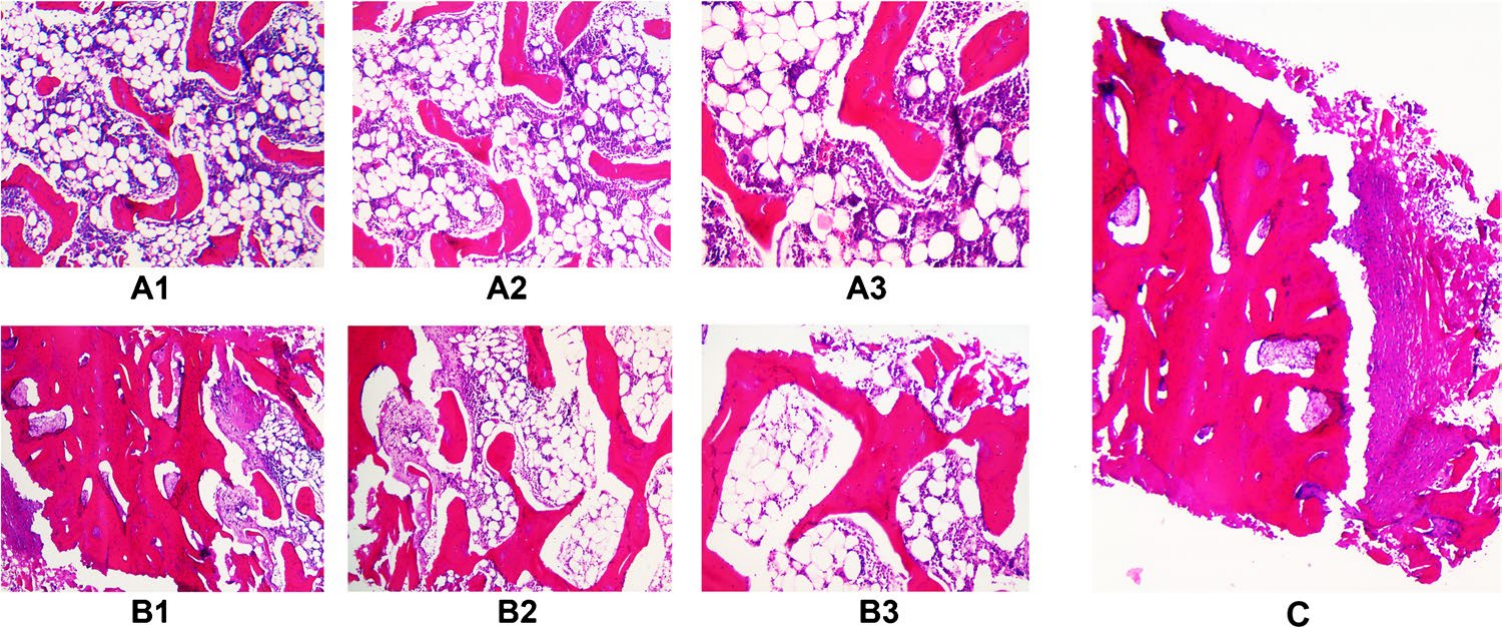
Histological images from the bone samples with haematoxylin and eosin (H&E) staining from the inner central spongiosa (trabecular bone, A1–3) and the spongiosa near the cortical bone (B1–3). The corresponding magnifications are 100 × (A1, B1), 50 × (A2, B2) and 25 × (A3, B3). (C) corresponds to a sample (25 ×) from the cutting edge of the drilling device at the transition zone from soft tissue to cortical bone

The obtained core specimens were subsequently seen by a pathologist (Fig. 5). After H&E staining, no signs of mechanical or thermal damage were found. The cortical bone as well as the central spongiosa and especially the cutting edge of the drilling device, where shearing energy, frictional effects and heat are most pronounced, appeared microscopically intact. Figure 5 shows histological sections from the core specimens including both the inner central spongiosa (A1–3) and the spongiosa near the cortical bone (B1–3) with magnifications of 100 × , 50 × and 25 × without any signs of drilling-related thermal damage such as thermal osteonecrosis.

## Discussion

Currently, there is a scarcity of in vivo data on heat generation and potential bone damage in battery-powered drill-assisted bone biopsies. After in vivo examination of the femur in pigs, we found that battery-powered, drill-assisted bone biopsies with an active drilling time of 20 s over a total observation period of 60 s showed only a low heating effect of a median of 0.1 °C and did not result in any micro- or macroscopic mechanical or thermal damage.

The bone has a microstructure with several interfaces, e.g. between marrow spaces and mineralized matrix or between mineralized matrix and fibrous tissue [13]. Heat generation follows from plastic deformation and friction adjacent to the drilling device and the bone interface [14]. Depending on the increase in temperature and the exposure time, thermal injuries may occur with changes in bone tissue properties and protein denaturation, desiccation and dehydration, resulting in cell death (thermal necrosis) [5, 15–18]. Thermal osteonecrosis is thus a direct consequence of the combined effects of increased temperature and the duration of increased temperature [16, 19]. In a study on rabbits, Lundskog et al. observed that a temperature of 55 °C applied for a duration of 30 s caused irreversible damage of bone cells [20]. Eriksson et al. performed a vital microscopic rabbit study and showed that bone tissue loses its function when heated to 47 °C for 1 m [21]. As none of these previous studies was carried out in vivo on human bone tissue, the exact threshold temperature for the death of human bone tissue is still unknown. However, the majority of researchers assume that a temperature of over 47 °C for 1 min can be considered a threshold for thermal osteonecrosis [22].

During bone drilling, the temperature and the level of stress in the bone tissue depend on the drilling parameters, which include speed, feed rate, energy, cooling and depth, with the majority of experimental examinations recommending high speed [22]. Technical advances in bone biopsy systems led to the development of battery-powered devices [23, 24]. Lee et al. reported battery-powered bone drilling using the same device to be safe and effective for biopsy sampling of focal bone lesions [3]. In addition, they observed a reduction of patient pain, shorter intervention time and lower radiation dose as well as an improved user control [3]. Similar results were reported by Berenson et al., who found that bone marrow samples were secured more rapidly and that patients experienced less intermediate-term pain [25]. The results of our porcine in vivo study furthermore suggest that there is no relevant heat development in the bone during drill-assisted bone biopsy sampling and that there is no apparent thermal tissue damage. A surprising finding of our study was a decrease in temperature immediately after drilling in two pigs. What we can imagine is that the drilling device was placed and inserted particularly quickly and easily in these two animals and therefore still had almost room temperature, which could have potentially led to a short-term cooling of the temperature probe.

The diagnostic yield of a bone biopsy can be affected by a multitude of factors. These include the histopathological architecture of the bone lesion, location of the lesion and the presence of mechanical artefacts and thermal osteonecrosis [4]. One limitation of our study is that we investigated a single percutaneous drilling device with a single speed and direction of rotation and a narrow range of possible needle sizes of 10 to 11gauge for bone access and 12 to 13 gauge for biopsy. Another limitation of the present study is that the drilling system was only investigated in healthy porcine bones and not in bones with lytic, sclerotic or suspected infectious lesions. Therefore, the results of the present study may not be directly transferable to humans with lytic or sclerotic bone lesions. Besides, the present study only investigated the effects of an active drilling time of 20 s. In the clinical settings, longer drilling times may be necessary in some patients. For such cases, no conclusion regarding possible temperature development can be drawn from our results. A further shortcoming of the present study is that drilling-induced heat generation was investigated in the living pig femur instead of a human femur, which differ in cortical and trabecular thickness. On the other hand, pigs are considered to be a good non-human model for a better understanding of the human skeleton, with similarities in terms of bone biology and bone mineral density [26][26]. Finally, it is important to note that our study design differs from applications in orthopaedic surgery, where bone drilling procedures are mainly used for bone reconstruction surgery and to insert implants.

## Conclusions

Overall, this preliminary study shows that battery-powered, drill-assisted harvesting of bone core specimens does not appear to cause mechanical or thermal damage, if the procedure is limited to an active drilling time of 20 s. As we only investigated normal bone tissue in pigs, the results may not be directly transferable to pathological bone lesions.

## Data Availability

All data are available within the manuscript and the supplementary material.
